# Integration of exercise and sports medicine curriculum in China: a structured pilot course evaluation conducted among medical students

**DOI:** 10.1186/s12909-026-09013-0

**Published:** 2026-05-09

**Authors:** Liu Guochun, Qing Chang, Ding Fu, Huang Xiaoxiao, Yan Shenglan, Luo Mengting, Yan Pingping, Yu Gao, Zhang Yu, Zhang Lingyun, Li jianyu, Guo Jia, Chen Xiaoke, Gao Fengwei, Ding Hao, Chen Peijie

**Affiliations:** 1 College of Exercise Medicine, Chongqing Medical University, Chongqing, China; 2 Division of Sports Science and Physical Education, Tsinghua University, Beijing, China; 3 Nursing Department of the First Affiliated Hospital of Chongqing Medical University, Chongqing, China; 4 College of Sports Health, Shanghai University of Sport, Shanghai, China

**Keywords:** Medical education, Sports and exercise medicine, Physical activity, Curriculum intervention, Chronic disease prevention

## Abstract

**Background:**

The integration of sports and exercise medicine curriculum (SEMc) including physical activity promotion and exercise prescription competencies into medical curricula is increasingly recognised within health profession education (HPE), yet evidence of its educational impact remains limited. This study evaluated the effects of a structured SEMc on medical students’ knowledge, skills, confidence, and attitudes in exercise prescription.

**Methods:**

Medical students (*n* = 10) completed a 4-week SEMc on the basis of international frameworks and China’s national guidelines; controls (*n* = 14) received no training. All participants completed baseline and post-course knowledge tests and a course-developed questionnaire with high internal consistency.The outcomes were analysed via repeated-measures ANOVA and t tests.

**Results:**

Participation in the SEMc significantly enhanced medical students’ competency in exercise prescription. Students who received training demonstrated substantial gains in knowledge, with scores improving from baseline and exceeding those of controls at post-assessment (95.2 ± 3.3 vs. 66.6 ± 17.1; *p* < 0.001). Improvements were sustained at the one-month follow-up, despite a slight decline from the immediate post-test (*p* = 0.041). The questionnaire data supported these findings, showing significant increases in skills (3.43 ± 0.67 to 4.18 ± 0.54; *p* = 0.030) and confidence (3.52 ± 0.55 to 4.07 ± 0.58; *p* = 0.046). Motivation remained consistently high (*p* = 0.290), and self-perceived knowledge did not change significantly (*p* = 0.170). Students not participating in the SEMc showed no notable changes (*p* > 0.10). At post-intervention, the SEMc group scored higher than controls in knowledge (*p* = 0.020), skills (*p* < 0.001), and confidence (*p* = 0.002).

**Conclusion:**

In this quasi-experimental pilot study, a brief, structured SEMc was associated with improvements in medical students’ exercise prescription–related knowledge, skills, and confidence. Embedding SEMc into mainstream curricula may equip future physicians with essential capacities for chronic disease prevention, aligning with the WHO’s Global Action Plan on Physical Activity.

**Graphical Abstract:**

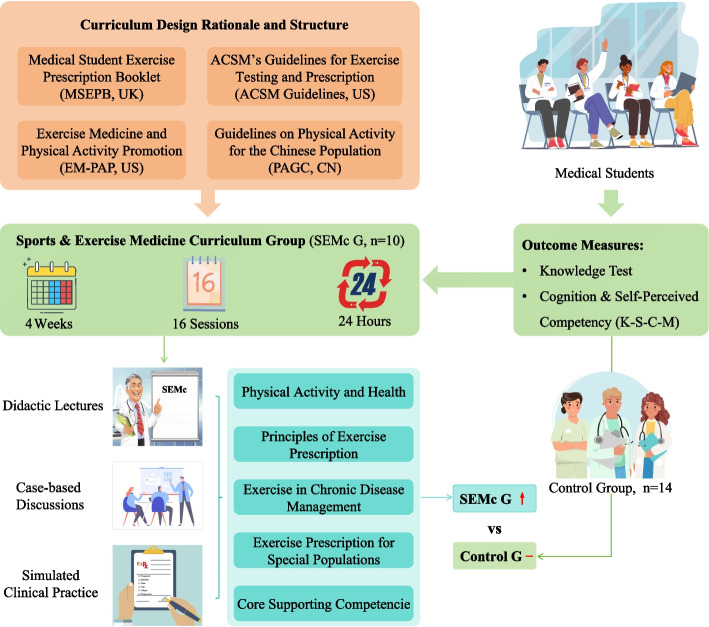

**Supplementary Information:**

The online version contains supplementary material available at 10.1186/s12909-026-09013-0.

## Background

During the paradigm shift in health profession education (HPE), competency-based training has been prioritised to meet the evolving demands of healthcare [1]. In parallel, integrating physical activity promotion and sports and exercise medicine (SEM) into undergraduate medical curricula has become a policy- and practice-relevant priority, supporting health promotion and chronic disease prevention and signalling a shift from disease-centred training toward health-oriented care [2, 3]. The World Health Organisation (WHO) Global Action Plan on Physical Activity 2018–2030 further underscores this need by calling for a whole-of-society approach and explicitly emphasising healthcare-sector action, including incorporation of physical activity into health services and workforce upskilling through education [4]. This position aligns with the *Global Competency and Outcomes Framework for Health Workforce Education*, which includes physical activity intervention as a core competency for health professionals [5]. However, medical curricula worldwide remain predominantly oriented toward pharmacological and procedural care, while SEM training is often fragmented, unstandardised, or weakly evidence-informed, leaving many graduates underprepared to deliver exercise-based interventions [6, 7].

To address this systemic shortfall, 26 countries have introduced physical activity-related competencies into dedicated courses or professional tracks at the undergraduate, postgraduate, and continuing education levels, thereby fostering the longitudinal development of medical students, residents, and sports medicine specialists [8]. For example, relevant curricula have been implemented in 83.87% of UK medical schools [9], 78.4% in the United States [10], 50% in Switzerland [11], and 88.2% in Australia [12]. The European Union is also advancing this agenda by forming interuniversity alliances to support physical activity promotion education within health professions training [2]. Amidst the increasing global burden of chronic disease, medical curricula are increasingly integrating exercise and health education to equip future physicians with core competencies in exercise prescription.

Despite recognition of physical activity as central to health promotion, SEM remains underrepresented in undergraduate curricula. In the United States, although 78.4% of medical schools include physical activity promotion and exercise prescription, total teaching averages ~ 8 h, and fewer than half of schools consider it adequate for clinical practice [10]. A similar situation has been observed in Australia. Although 88.2% of Australian medical schools incorporate specific content on physical activity [12], many fail to include instruction on national or international physical activity guidelines. Only 46.7% of schools teach recommendations related to exercise training, and the total instructional time ranges from 6.6–12.4 h across 4– to 6-year programs [11]. In Switzerland, SEM integration is minimal; although only half of students recognise its role in noncommunicable disease management, 95% express demand for formal training [10]. The United Kingdom has positioned itself at the forefront of addressing this educational deficit. Lancaster Medical School pioneered the integration of the Movement for Movement Resources into its undergraduate curriculum, embedding physical activity as a core clinical competency [13]. Complementing this initiative, the UK Faculty of Sport and Exercise Medicine developed a self-directed manual tested in students from 22 medical schools, which improved adult physical activity guideline knowledge and prescribing confidence, with correct case responses increasing by 32% (osteoarthritis) and 44% (cancer) post-intervention [14]. Together, these data suggest that the key challenge is shifting from nominal coverage to structured, guideline-aligned, and assessable SEM training.

In China, SEM education is nascent and fragmented, delivered mainly through (1) physical education institutions emphasising rehabilitation and sports injuries, (2) voluntary certification programmes led by the Chinese Association of Sports Science, and (3) limited integration into undergraduate medical curricula, with Chongqing Medical University as an early exemplar [15]. Despite the Healthy China 2030 strategy increasing physical activity as a health priority, progress has largely remained conceptual and rhetorical rather than being operationalised, with no coherent framework across the educational continuum [16]. Currently, China lacks a comprehensive SEM education framework that spans undergraduate, postgraduate, and continuing medical education levels. Aligned with the WHO *Global Action Plan on Physical Activity 2018–2030* and China’s “*New Medicine*” reform, this quasi-experimental study evaluates the feasibility and educational impact of a structured SEM curriculum (SEMc). By comparing SEMc-trained students with controls, we aim to generate evidence to inform scalable integration of SEM within China’s medical education system.

### Intervention (SEMc): development and content

The curriculum was conceptualised through the integration of internationally recognised frameworks in SEM [17], ensuring both global coherence and contextual adaptability. In the United Kingdom, the “Movement for Movement” initiative and the *Medical Student Exercise Prescription Booklet* established by the Faculty of Sport and Exercise Medicine have served as pivotal models for embedding physical activity promotion into undergraduate education [18]. Building upon these foundations, the first national undergraduate SEM syllabus was recently formalised through a Delphi consensus, identifying 48 learning objectives organised into nine domains that span physical activity and health, exercise prescription, injury management, anti-doping, and care of special populations [19]. Parallel developments in North America, particularly the *ACSM’s Guidelines for Exercise Testing and Prescription* (11th ed.) and the American Medical Society for Sports Medicine (AMSSM) Exercise Medicine and Physical Activity Promotion (EM-PAP) framework, further reinforce the imperative to integrate SEM competencies into medical training without increasing curricular burden [2, 20]. Endorsements by allied professional bodies highlight the transnational relevance of these educational standards. While such frameworks provide robust international precedents, integration into Chinese undergraduate medical education has historically been limited, underscoring the need for a contextualised curriculum model.

To ensure cultural and clinical resonance, these international frameworks were synthesised with the *Guidelines on Physical Activity for the Chinese Population (2021)*, aligning the curriculum with national public health priorities [21]. This integration informed the development of a five-module SEMc that scaffolds learning from foundational mechanisms to applied clinical competencies (Table 1), with exercise prescription principles (FITT, safety, risk stratification, and individualisation) serving as the bridge between core science and condition-specific management. Across modules, exercise is positioned as a clinically actionable intervention for major chronic diseases, consistent with established epidemiological evidence linking physical activity to reduced morbidity and improved outcomes [13, 22–24]. To support transfer beyond knowledge acquisition, SEMc embeds active learning (case-based tasks, peer role-play, and structured feedback) and incorporates formative and summative assessments to evaluate knowledge, clinical reasoning, and readiness for practice.

**Table 1:** Core modules and key content of the SEMc

SEMc Curriculum Module	Key Content and Instructional Design
Physical Activity and Health	Mechanisms of action of physical activity on cardiopulmonary and musculoskeletal systems; associated health benefits and risk reduction; interpretation of WHO physical activity guidelines and the 2021 Chinese guidelines; global trends in physical activity
Principles of Exercise Prescription	The “FITT-VP” principles (Frequency, Intensity, Time, Type, volume and Progression); standardised aerobic and resistance training prescriptions; risk stratification and safety considerations
Exercise in Chronic Disease Management	Preventive and therapeutic exercise strategies for common chronic conditions (e.g., cardiovascular disease, type 2 diabetes, obesity, cancer); underlying physiological mechanisms and supporting evidence; case-based application of exercise prescriptions
Exercise Prescription for Special Populations	Principles of exercise prescription for older adults, children and adolescents, pregnant and postpartum women; functional characteristics and adaptive considerations; tailored exercise plans and precautions
Core Supporting Competencies	Strategies to enhance patient adherence and behavioural change strategies; critical appraisal of SEM literature; basics of research and experimental design

## Methods

### Course intervention protocol

**Fig. 1:**
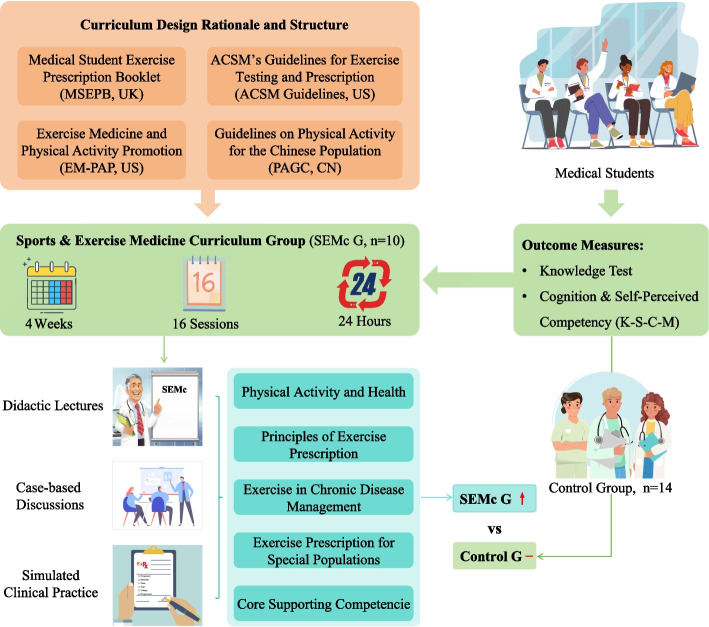
Schematic overview of the study design and intervention

This prospective educational intervention was conducted in China and approved by the Ethics Committee of Chongqing Medical University (2025–044). All participants provided written informed consent after receiving information on the course objectives, curriculum, assessment procedures, feedback processes, data protection measures, and right to withdraw at any time without penalty. The study design is depicted in Fig. 1. The SEMc group (SEMc G) was structured as follows: 4-week SEMc was delivered face-to-face (16 sessions; four per week; 1.5 h per session; 24 h in total). Instructions employed didactic lectures, case-based discussions, and supervised practical exercises led by faculty with expertise in sports medicine. Learners apply competencies by developing individualised exercise prescriptions in simulated clinical scenarios. The control group (CG) received no SEM-related instructions during the study period.

### Participants

A total of 24 undergraduate students from Chongqing Medical University participated in the study (Table 2). The SEMc G comprised 10 students enrolled in the university’s SEMc, who received the full SEMc as described. The CG included 14 students who were recruited through a university WeChat group and did not receive any SEM-related instruction during the study period.

**Table 2:** Demographic characteristics of the participants

Group	n	Female %	Mean Age	Academic Majors
SEMc G	10	90.00	20.6	Nursing, Clinical Medicine, Psychiatry
C G	14	78.60	18.14	Preventive Medicine, Clinical Medicine, Nursing, Public Health, Psychiatry, Medical Imaging, Medical Laboratory Science

*SEMc* Sports and Exercise Medicine curriculum

### Outcome measures

A custom-designed written test was developed to assess students’ mastery of SEM core concepts on the basis of the course learning objectives. The test included multiple-choice, true/false, and short-answer items, with a total score of 100 points. Content validity was reviewed and approved by two experts in sports medicine. SEMc G completed the knowledge test at three time points: precourse (T1), immediately post-course (T2), and one month post-course (T3). The CG completed the test at two matched time points: T1 and T3. The examination papers were developed specifically for this study and are provided as a supplementary file (see Additional file 1:SEM Curriculum Examination Papers, Versions A and B).

Cognition and self-perceived competency A structured, self-administered questionnaire was used to evaluate students’ perceived competency in exercise prescription across four domains.The questionnaire was also developed by our course team specifically for this study (see Additional file 2: Survey on the Learning Outcomes of the Sports and Exercise Medicine).

- Knowledge (K): Understanding key SEM concepts and guidelines.
- Skills (S): Ability to design and implement individualised exercise prescriptions.
- Confidence (C): Self-efficacy in applying exercise interventions in clinical contexts.
- Motivation & Attitudes (M): Commitment to integrating exercise into preventive and therapeutic practices.

Each domain consists of several items rated on a 5-point Likert scale (1 = strongly disagree, 5 = strongly agree). Domain scores were calculated as the means of the respective items, and an overall composite score was derived by averaging across all four domains. The questionnaire demonstrated strong internal consistency across both groups and time points: for the SEMc G, Cronbach’s α was 0.952 (T1) and 0.938 (T2), and for the CG, Cronbach’s α was 0.963 (T1) and 0.902 (T2). All the responses were collected anonymously via the Wenjuanxing online platform. Both groups completed the questionnaire one week prior to and one week after the intervention period.

### Statistical analysis

Analyses were performed in SPSS 26.0. Baseline differences were tested between groups. Knowledge outcomes were examined with mixed-design ANOVA (group × time), with paired-samples t tests for within-group contrasts and independent-samples t tests for between-group contrasts. The questionnaire data were analysed via paired- and independent-samples t tests. All tests were two-tailed (α = 0.05). Effect sizes were calculated as Cohen’s d values. Data are presented as the means ± SDs.

## Results

### Baseline equivalence between the intervention and control groups

Baseline characteristics are summarised in Table 3. Preintervention knowledge scores were higher in the SEMc G (76.4 ± 9.3) than in the CG (65.7 ± 15.8). Although the between-group comparison yielded t(22) = 2.08, *p* = 0.05, indicating a borderline imbalance in baseline knowledge at study entry. No significant between-group differences were observed across the questionnaire domains (knowledge: *p* = 0.85; skills: *p* = 0.20; confidence: *p* = 0.11; motivation & attitudes: *p* = 0.96), suggesting that both groups had similar self-perceived competence prior to the intervention.

**Table 3:** Baseline comparison between the SEMc G and the CG

Outcome Measure	SEMc G (*n* = 10), mean ± SD	CG (*n* = 14), mean ± SD	*t value*	*p value*
Knowledge test score (0–100)	76.4 ± 9.3	65.7 ± 15.8	t(22) = 2.08	0.05
Knowledge domain (1–5 scale)	3.88 ± 0.76	3.74 ± 0.59	NA	0.85
Skills domain (1–5 scale)	3.43 ± 0.67	2.38 ± 0.85	NA	0.20
Confidence domain (1–5 scale)	3.52 ± 0.55	2.63 ± 1.09	NA	0.11
Motivation & attitudes (1–5 scale)	3.98 ± 0.75	4.39 ± 0.57	NA	0.96

The values are presented as the means ± SDs. Between-group differences were analysed via independent-samples t tests

*NA* Not applicable where test statistics were not calculated, *SEMc* Sports and Exercise Medicine curriculum, *SD* Standard deviation

### Effects of the SEMc intervention

A mixed-design repeated-measures ANOVA demonstrated a significant main effect of time (F = 30.21, *p* < 0.001) and a significant time × group interaction (F = 22.45, *p* < 0.001), indicating that changes in knowledge scores over time differed between groups (Table 4).

**Table 4:** Within- and between-group comparisons of knowledge test scores in the SEMc and CG

Comparison	Time1, Mean ± SD	Time2, Mean ± SD	Mean Difference	t(df)	*p value*	Cohen’s d
SEMc G: Pre vs Post	76.4 ± 9.3	95.2 ± 3.3	18.8	7.04 (9)	< 0.001 ↑	2.23
SEMc G: Pre vs 1-month Follow-up	76.4 ± 9.3	87.2 ± 10.6	10.8	2.89 (9)	0.018 ↑	0.91
SEMc G: Post vs 1-month Follow-up	95.2 ± 3.3	87.2 ± 10.6	﹣8.0	﹣2.38 (9)	0.041 ↓	–0.75
CG: Pre vs 4 weeks later	65.7 ± 15.8	66.6 ± 17.1	0.9	0.14 (13)	0.894	0.06
post-test: SEMc G vs CG	95.2 ± 3.3	66.6 ± 17.1	28.6	5.19 (22)	< 0.001 ↑	2.15

SEMc G post-test, immediate assessment after course completion; CG follow-up, same time point after 4 weeks of routine study without SEMc. All *p* values are two-tailed. *SD* Standard deviation. The arrows (↑/↓) indicate significant differences

In the SEMc G, the mean scores rose from 76.4 ± 9.3 at baseline to 95.2 ± 3.3 immediately post-course, a large and statistically robust improvement (*t*(9) = 7.04, *p* < 0.001, *d* = 2.23). This reflects a statistically significant improvement in knowledge immediately following the intervention. At one month, the score declined to 87.2 ± 10.6 but remained significantly above baseline (*t*(9) = 2.89, *p* = 0.018, *d* = 0.91), indicating meaningful retention despite some attrition (post vs follow-up: *t*(9) = ﹣2.38, *p* = 0.041, *d* = ﹣0.75). In contrast, the CG exhibited no significant change across the same period (65.7 ± 15.8 vs. 66.6 ± 17.1, *t*(13) = 0.14, *p* = 0.894, *d* = 0.06), indicating stability of knowledge scores under routine medical education.

Between-group comparisons showed that preintervention knowledge scores were higher in the SEMc group than in the control group (t(22) = 2.08, *p* = 0.05), indicating a borderline imbalance at study entry. Post-test scores in the SEMc group (95.2 ± 3.3) were significantly higher than those in the control group (66.6 ± 17.1; t(22) = 5.19, *p* < 0.001, d = 2.15). Overall, SEMc participation was associated with significant short-term knowledge gains with partial retention at one-month follow-up, whereas no comparable changes were observed in the control group.

### Increased self-perceived competency in exercise prescription following SEMc

The influence of SEMc training on self-perceived competence across four domains (knowledge, skills, confidence, and motivation/attitudes) is summarised in Table 5. Baseline questionnaire domain scores did not differ significantly between groups. Following the 4-week SEMc training, students showed significant improvements in skills (3.43 ± 0.67 to 4.18 ± 0.54; t(9) = 2.62, *p* = 0.03) and confidence (3.52 ± 0.55 to 4.07 ± 0.58; t(9) = 2.31, *p* = 0.046). The knowledge domain increased from 3.88 ± 0.76 to 4.32 ± 0.61, but the change was not statistically significant (*p* = 0.17). Motivation/attitudes scores were high at baseline (3.98 ± 0.75) and showed a small, non-significant increase to 4.33 ± 0.71 (*p* = 0.29), suggesting limited room for improvement.

**Table 5:** Within-group effects of a 4-week SEMc on knowledge, skills, confidence, and motivation compared with controls

Domain	SEMc G Pre, Mean ± SD	SEMc G Post, Mean ± SD	*t*(9)	*p value*	CG Pre, Mean ± SD	CG Post, Mean ± SD	*t*(13)	*p value*
Knowledge (K)	3.88 ± 0.76	4.32 ± 0.61	1.48	0.17	3.74 ± 0.59	3.77 ± 0.47	0.19	0.854
Skills (S)	3.43 ± 0.67	4.18 ± 0.54	2.62	0.03	2.38 ± 0.85	2.75 ± 0.54	1.35	0.2
Confidence (C)	3.52 ± 0.55	4.07 ± 0.58	2.31	0.046	2.63 ± 1.09	3.17 ± 0.85	1.73	0.108
Attitude & Motivation (M)	3.98 ± 0.75	4.33 ± 0.71	1.12	0.29	4.39 ± 0.57	4.38 ± 0.61	﹣0.05	0.96
Overall Mean Score	3.70 ± 0.55	4.22 ± 0.50	3.31	0.009	3.28 ± 0.71	3.52 ± 0.64	1.66	0.12

In contrast, the CG, which received no SEMc instruction, exhibited no significant pre–post changes across any domain (knowledge: *p* = 0.854; skills: *p* = 0.20; confidence: *p* = 0.108; attitude: *p* = 0.96), suggesting that scores in the control group remained stable under standard medical education. Between-group analyses confirmed the superiority of the SEMc intervention (Table 6). During the post-test, SEMc participants outperformed controls in terms of knowledge (4.32 ± 0.61 vs. 3.77 ± 0.47; t(22) = 2.46, *p* = 0.022), skills (4.18 ± 0.54 vs. 2.75 ± 0.54; t(22) = 6.42, *p* < 0.001; Cohen’s d = 2.66), and confidence (4.07 ± 0.58 vs. 3.17 ± 0.85; t(22) = 3.48, *p* = 0.002; d = 1.42). The effect sizes were large, indicating that the observed differences were not only statistically significant but also substantial in magnitude.

**Table 6:** Postintervention between-group differences in competency outcomes

Domain	SEMc G Post, Mean ± SD	CG Post, Mean ± SD	*t*(22)	*p value*	Cohen’s *d*
Knowledge (K)	4.32 ± 0.61	3.77 ± 0.47	2.46	0.022	1.02
Skills (S)	4.18 ± 0.54	2.75 ± 0.54	6.42	< 0.001	2.66
Confidence (C)	4.07 ± 0.58	3.17 ± 0.85	3.48	0.002	1.42
Attitude & Motivation (M)	4.33 ± 0.71	4.38 ± 0.61	﹣0.05	0.96	﹣0.07
Overall Mean Score	4.22 ± 0.50	3.52 ± 0.64	3.18	0.004	1.3

Attitudes and motivation remained high in both groups (SEMc: 4.33 ± 0.71; control: 4.38 ± 0.61; *p* = 0.960), indicating that both groups shared consistently positive views toward exercise prescription, and a ceiling effect that limited observable gains in this domain. The SEMc G’s composite competency score rose significantly from 3.70 ± 0.55 to 4.22 ± 0.50 (t(9) = 3.31, *p* = 0.009), whereas the CG showed no statistically significant change (from 3.28 ± 0.71 to 3.52 ± 0.64, *p* = 0.12). These findings reinforce the overall effectiveness of the course in improving students’ cognitive readiness for integrating exercise-based interventions into clinical care.

## Discussion

This quasi-experimental evaluation indicates that a brief, structured SEMc delivered over four weeks can produce meaningful gains in medical students’ SEM-related learning outcomes [25]. Compared with controls, SEMc participants showed substantial improvement in written knowledge immediately post-course, with significant retention at one-month follow-up. SEMc training was also associated with improvements in self-perceived exercise-prescription competence, particularly skills and confidence, whereas attitudes and motivation were already high at baseline and showed limited room for change [10, 26, 27]. These findings support the feasibility of embedding SEM competencies, including physical activity promotion and exercise prescription, within crowded undergraduate curricula through a competency-oriented design that prioritises authentic tasks and feedback [14]. This approach is consistent with international calls, including the International Olympic Committee consensus, to treat SEM competencies as essential components of the medical graduate toolkit, and it offers an implementable model that can deliver measurable educational gains without substantial increases in contact hours [28–31].

At the same time, strengthening physicians’ SEM competencies should be viewed as complementary to, rather than a substitute for, team-based delivery of exercise and physical activity interventions. The WHO has highlighted interprofessional education and collaborative practice as important strategies to strengthen health systems and improve outcomes [32]. In several health systems, credentialed clinical exercise professionals, including clinical exercise physiologists and accredited exercise physiologists, contribute specialist assessment, risk stratification, exercise prescription, supervision, and behaviour-change support for people living with chronic conditions [33, 34]. From this perspective, SEMc is intended to equip future physicians to initiate evidence-informed counselling, identify appropriate candidates for referral, and collaborate effectively with clinical exercise professionals within a coordinated care pathway.

### Knowledge gains and short-term retention

The efficacy of deep learning extends beyond mere knowledge transmission; it hinges on how information is organised, the authenticity of the learning context, and the frequency with which knowledge is revisited and applied [35]. Accordingly, SEMc was designed to activate encoding and retrieval through problem-based learning, case-driven discussions, and collaborative prescription workshops. These methods did not merely supplement content delivery, they fundamentally restructured the learning experience to support durable cognitive imprinting. This design aligns with principles of constructive alignment, whereby intended learning outcomes, learning activities, and assessment are intentionally mapped to reinforce the same competencies. In SEMc, clinically relevant outcomes (e.g., individualised exercise prescription and risk-informed decision-making) were paired with authentic learning tasks and matched assessments, which likely reduced the gap between declarative knowledge and procedural application. Such active and practice-oriented approaches have been associated with improved learner engagement and performance in medical education compared with predominantly passive formats. Repeated, feedback-rich practice in simulated clinical tasks may also reflect deliberate practice principles, consistent with the observed improvements in skills and confidence [36, 37]. The observed knowledge gains suggest that the instructional design may have engaged deeper cognitive processes, consistent with schema integration and rehearsal mechanisms. Crucially, retention effects remained statistically significant at the one-month follow-up, implying that short-term instruction if cognitively aligned can seed medium-term knowledge consolidation.

However, the modest decline in scores over time also reflects the expected operation of cognitive decay mechanisms in the absence of reinforcement. This underscores a well-established tenet in educational theory: learning that is not reactivated or contextually reapplied is vulnerable to attrition. Addressing this challenge requires a longitudinal instructional architecture in which SEM is embedded across curricular phases, ideally through vertically integrated clinical exposures and rotation-based application pathways. As highlighted in recent competency-based curricular proposals [2], sustained cognitive gains in exercise medicine are best achieved when instruction is scaffolded by practice-oriented modules that reconnect theoretical principles to authentic clinical reasoning tasks. By constructing a recursive cognitive circuit linking instruction, application, and recognition the educational impact of SEM training can move beyond short-term achievement to long-term professional competency.

### Skills/confidence outcomes and practical performance

The observed enhancement in exercise prescription proficiency and clinical application confidence should not be interpreted simply as a linear “input–output” effect of instructional exposure. Rather, it reflects a dual-process mechanism: the intentional facilitation of skill transfer and the cultivation of self-efficacy. This SEMc strategically employs immersive pedagogies, including simulated case scenarios, role-play exercises, and peer critique, to anchor cognitive learning within authentic clinical contexts. These experiences enabled the students to reconfigure the linkage between declarative knowledge and procedural behaviour within situated action, thereby transforming competence into a confident application. The observed skill improvement was substantial, and appears comparable to or greater than those reported in some prior medical education interventions [11, 38], underscoring not only the instructional effectiveness but also the pedagogical specificity with which the learning experience was engineered. By engaging kinesthetic memory and contextual reasoning, the course stimulated the neurocognitive substrates underlying professional performance readiness.

This finding is particularly salient given the prevailing global deficit in structured training for physical activity prescription. For example, only 3% of medical students in the UK report formal instruction in this area, and fewer than 20% of U.S. medical schools offer systematic curricular coverage [10]. In our study, skill and confidence improvements were achieved within a constrained timetable, suggesting that curriculum integration may be feasible when learning activities are tightly aligned with clinically relevant competencies.

### Attitudes/motivation and ceiling effects

The absence of a significant change in the attitudes/motivation domain likely reflects a ceiling effect, as baseline endorsement was already high. When baseline scores are high, additional short-term curricular exposure may yield limited observable change in self-reported attitudes. This pattern suggests that, in this cohort, the intervention primarily influenced knowledge- and skill-related domains rather than shifting pre-existing attitudes. This phenomenon is not confined to our sample but resonates with findings from broader international cohorts. For example, a national survey of 1,764 Swiss medical students revealed that 95% support the inclusion of SEM in undergraduate training [10], whereas UK students have consistently endorsed physical activity as a frontline therapeutic tool [8]. These data collectively suggest that positive disposition toward SEM is no longer a barrier to educational integration; rather, it is an existing asset to be leveraged.

The educational implications are clear: when attitudinal readiness is already high, instructional resources are more effectively allocated toward domains with greater potential for cognitive consolidation or behavioural transformation. Specifically, curricular interventions should pivot from “awareness raising” to competence building and behavioural enactment. This shift aligns with evidence from clinical practice settings, where gaps remain not in belief but in confidence and the ability to implement physical activity interventions [39]. Thus, future SEM education should treat attitudinal congruence as a strategic launchpad rather than a target outcome—capitalising on student enthusiasm to deepen practical engagement, reinforce self-efficacy, and facilitate the translation of training into clinical routines.

### Implementation implications for curriculum integration

This study demonstrates that even with limited time and resources, a strategically integrated SEMc can rapidly improve medical students' knowledge, competence, and self-efficacy. These findings support repositioning exercise medicine from an optional topic to a core clinical competency [29]. To address curricular crowding, SEMc should be embedded as an integrated competency thread rather than a standalone block. Learning outcomes can be mapped onto existing system-based teaching (e.g., cardiometabolic, musculoskeletal, mental health) and primary care clerkships, while skills training can be incorporated into clinical skills sessions and OSCEs—including physical activity history taking, risk screening, and exercise prescription. Core knowledge can be delivered asynchronously via e-learning, preserving face-to-face time for practice and feedback. This "embed rather than add" strategy aligns with international guidelines on physical activity education and WHO recommendations to strengthen preservice health professional training [3, 14, 40].

When integrating the concept of promoting physical activity into the routine clinical practice in China (such as sarcopenia, hypertension, diabetes, obesity, etc.), it is very important to take into account the background and cultural factors [41, 42]. Some patients may be concerned about potential safety issues of exercise and will prioritize rest treatment for chronic diseases [43]. To enhance local relevance, SEMc was aligned with the “Chinese Physical Activity Guidelines (2021)”, and emphasized risk stratification, staged progression, and practical, easy-to-follow prescriptions for common chronic diseases in the Chinese clinical environment [44]. Role-playing and case-based sessions were also used to practice patient-centered explanations to address safety issues and support shared decision-making. These features work together to aim not only at enhancing the knowledge that students acquire, but also at improving their ability to promote physical activity in the familiar cultural nursing environment and how to communicate and implement it.

The global epidemiological transition toward lifestyle-related diseases has redefined the boundaries of medical responsibility, demanding that physicians not only manage disease but also actively engage in its prevention. Within this paradigm shift, SEM education has emerged as a critical interface linking evidence-based prevention strategies with everyday clinical practice. As articulated in the WHO Global Action Plan on Physical Activity (2018–2030), there is a pressing need to embed physical activity competencies into both preservice and in-service health professional training to address the systemic inertia surrounding inactivity-related morbidity [4]. More recently, the 75th World Health Assembly (2022) reiterated this call, identifying the capacity-building of health personnel in physical activity counselling and chronic disease prevention as a strategic priority for all member states (WHO, WHA75/2022/REC/1). From a curricular perspective, this underscores the urgent need to integrate SEM into the medical school core—not as an elective but as a longitudinal thread that scaffolds theoretical grounding, applied skills, and behavioural implementation [45]. Empirical evidence further substantiates this trajectory. Physical activity has been shown to reduce the incidence and severity of cardiovascular disease, diabetes, cancer, and depression, among other NCDs [27]. However, these population-level benefits remain unrealised if frontline clinicians lack the knowledge or confidence to initiate exercise prescriptions. Indeed, the absence of physician-driven exercise counselling often translates to missed opportunities for preventive intervention.

Taken together, the observed gains in knowledge, skills, and confidence suggest that SEMc may help prepare future physicians to initiate evidence-informed counselling and exercise prescription in routine care. This pedagogical orientation aligns closely with the United Nations Sustainable Development Goal 3.4, which calls for a one-third reduction in premature mortality from NCDs by 2030 through prevention and treatment [46]. To this end, medical schools must act not as passive transmitters of biomedical knowledge but as incubators of preventive medicine capacity [47]. However, this pilot evaluation cannot determine whether these educational gains translate into sustained clinical behaviour change or patient outcomes. Future studies should use stronger objective performance measures and longer follow-up to evaluate transfer to practice.

## Conclusion

This study provides preliminary evidence supporting the necessity and potential effectiveness of incorporating SEM into undergraduate medical education. The curricular intervention significantly improved medical students’ knowledge and skills. This pilot project offers insights for medical education management in Chinese medical schools and suggests that global health policy frameworks can be adapted to local contexts. These findings align with the paradigm shift in medical education from being “disease-centered” to “health-oriented” and highlight SEM as a promising avenue within HPE that links clinical competence development, health promotion, and interprofessional collaboration. In the future, more medical schools should be encouraged to adopt and refine similar programs to cultivate a new generation of physicians who are not only proficient in clinical diagnosis and treatment but also capable of leading lifestyle-based prevention and chronic disease management.

### Limitations

This study has several limitations. First, the small sample size and single-site design may limit generalizability. Second, the nonrandomised group allocation, raises the possibility of unmeasured confounding. Third, the short intervention duration and one-month follow-up precluded the assessment of long-term knowledge retention or clinical application. Future studies should adopt randomised designs, expand sample diversity, standardise evaluation tools, and include extended follow-up to assess the sustained impact on clinical competence.

## Supplementary Information

Supplementary Material 1.

Supplementary Material 2.

## Data Availability

Data are provided within the manuscript or supplementary information files.

## References

[1] Abdalla ME, Taha MH, Onchonga D, et al. Instilling social accountability into the health professions education curriculum with international case studies: AMEE guide no. 175. Med Teach. 2024;47(7):1083–96. 10.1080/0142159X.2024.2412098. 39418524 10.1080/0142159X.2024.2412098

[2] Frenk J, Chen L, Bhutta ZA, et al. Health professionals for a new century: transforming education to strengthen health systems in an interdependent world. Lancet. 2010;376(9756):1923–58. 10.1016/S0140-6736(10)61854-5. 21112623 10.1016/S0140-6736(10)61854-5

[3] Asif I, Thornton JS, Carek S, et al. Exercise medicine and physical activity promotion: core curricula for US medical schools, residencies and sports medicine fellowships: developed by the American Medical Society for Sports Medicine and endorsed by the Canadian Academy of Sport and Exercise Medicine. Br J Sports Med. 2022;56(7):369–75. 10.1136/bjsports-2021-104819. 35012931 10.1136/bjsports-2021-104819

[4] World Health Organization. Global action plan on physical activity 2018–2030: more active people for a healthier world. Geneva: WHO; 2018.

[5] World Health Organization, World Federation of Public Health Associations. Global competency and outcomes framework for health workforce education. Geneva: World Health Organization; 2015. Available from: https://iris.who.int/handle/10665/330091. Accessed 11 Jun 2024.

[6] Metsios GS, Morres ID, Fatouros I, et al. Implementation of physical activity in the curricula of medical schools and healthcare professions across Europe: the VANGUARD project study protocol. Mediterr J Rheumatol. 2024;35(3):498–503. 10.31138/mjr.290724.iac. 39463863 10.31138/mjr.290724.iac PMC11500127

[7] Sousa JR, Afreixo V, Carvalho J, Silva P. Nutrition and physical activity education in medical school: a narrative review. Nutrients. 2024;16(16):2809. 10.3390/nu16162809. 39203945 10.3390/nu16162809 PMC11357297

[8] Weiler R, Chew S, Coombs N, Hamer M, Stamatakis E. Physical activity education in the undergraduate curricula of all UK medical schools: are tomorrow’s doctors equipped to follow clinical guidelines? Br J Sports Med. 2012;46(14):1024–6. 10.1136/bjsports-2012-091380. 22846233 10.1136/bjsports-2012-091380 PMC3856633

[9] Gates AB, Swainson MG, Moffatt F, Kerry R, Metsios GS, Ritchie I. Undergraduate examination and assessment of knowledge and skills is crucial in capacity planning for the future healthcare workforce in physical activity interventions. Br J Sports Med. 2020;54(17):1015–6. 10.1136/bjsports-2019-101646. 31937575 10.1136/bjsports-2019-101646

[10] Stoutenberg M, Stasi S, Stamatakis E, et al. Physical activity training in US medical schools: preparing future physicians to engage in primary prevention. Phys Sportsmed. 2015;43(4):388–94. 10.1080/00913847.2015.1084868. 26365470 10.1080/00913847.2015.1084868

[11] Carrard J, Pandya T, Niederhauser L, Infanger D, Schmidt-Trucksaess A, Kriemler S. Should sports and exercise medicine be taught in the Swiss undergraduate medical curricula? A survey among 1764 Swiss medical students. BMJ Open Sport Exerc Med. 2019;5(1):e000575. 10.1136/bmjsem-2019-000575. 10.1136/bmjsem-2019-000575 PMC6733322 31548904

[12] Strong A, Stoutenberg M, Hobson-Powell A, Hargreaves M, Beeler H, Stamatakis E. An evaluation of physical activity training in Australian medical school curricula. J Sci Med Sport. 2017;20(6):534–8. 10.1016/j.jsams.2016.10.011. 28209318 10.1016/j.jsams.2016.10.011

[13] Gates AB, Swainson MG, Isba R, Wheatley RG, Curtis FA. Movement for Movement: a practical insight into embedding physical activity into the undergraduate medical curriculum exemplified by Lancaster Medical School. Br J Sports Med. 2019;53(10):609–10. 10.1136/bjsports-2018-100243. 30482781 10.1136/bjsports-2018-100243

[14] Pugh G, O’Halloran P, Blakey L, Leaver H, Angioi M. Integrating physical activity promotion into UK medical school curricula: testing the feasibility of an educational tool developed by the Faculty of Sports and Exercise Medicine. BMJ Open Sport Exerc Med. 2020;6(1):e000679. 10.1136/bmjsem-2019-000679. 10.1136/bmjsem-2019-000679 PMC7279672 32547778

[15] Liu G, Cao C. Construction of professional, occupational and employment systems for the integration of sports and medicine in China: overseas experience in clinical exercise physiology. Chin Sport Sci. 2022;42(12):29–42. 10.16469/j.css.202212004.

[16] Liu G, Wang Z, Cao C. From the concept of sports and medicine integration to the concrealisation of service models: international sports referral experience and its implications. Chin Sport Sci. 2024;44(07):35–50. 10.16469/J.css.2024KX007.

[17] Gaffney CJ. The genesis of a new sports and exercise science degree. Physiol News. 2018;(110):Spring. Available from: https://www.physoc.org/magazine-articles/the-genesis-of-a-new-sports-and-exercise-science-degree-2/. Accessed 22 Jul 2025.

[18] Faculty of Sport and Exercise Medicine UK (FSEM). Exercise prescription in health and disease: a series of cases for medical students. London: FSEM; 2024. Available from: https://www.fsem.ac.uk/wp-content/uploads/2024/06/Exercise-Prescription-Booklet.pdf. Accessed 22 Jul 2025.

[19] Vishnubala D, Iqbal A, Marino K, et al. Creating a sport and exercise medicine undergraduate syllabus: a delphi study. BMC Med Educ. 2023;23(1):179. 10.1186/s12909-023-04139-x. 36959591 10.1186/s12909-023-04139-x PMC10035170

[20] Frommeyer TC, Brittain GV, Wu T, Frommeyer D, Bett ES. Physical activity prescriptions: addressing a major gap in medical education. Med Sci Educ. 2025. 10.1007/s40670-025-02382-z. 10.1007/s40670-025-02382-z PMC12532528 41112883

[21] Guideline Committee for Physical Activity in Chinese Population. Guidelines on physical activity for Chinese population (2021). Chin J Public Health. 2022;38(2):145–51.

[22] Sprys-Tellner T, Levine D, Kagzi A. The application of exercise prescription education in medical training. J Med Educ Curric Dev. 2023;10:23821205231217892. 10.1177/23821205231217893. 38033378 10.1177/23821205231217893 PMC10685795

[23] Lee IM, Shiroma EJ, Lobelo F, et al. Effect of physical inactivity on major noncommunicable diseases worldwide: an analysis of burden of disease and life expectancy. Lancet. 2012;380(9838):219–29. 10.1016/S0140-6736(12)61031-9. 22818936 10.1016/S0140-6736(12)61031-9 PMC3645500

[24] Wahid A, Manek N, Nichols M, et al. Quantifying the association between physical activity and cardiovascular disease and diabetes: a systematic review and meta-analysis. J Am Heart Assoc. 2016;5(9):e002495. 10.1161/JAHA.115.002495. 27628572 10.1161/JAHA.115.002495 PMC5079002

[25] Buckler DG. General practitioners’ training for, interest in, and knowledge of sports medicine and its organisations. Br J Sports Med. 1999;33(5):360–4. 10.1136/bjsm.33.5.360. 10522642 10.1136/bjsm.33.5.360 PMC1756200

[26] Jaques R, Loosemore M. Sports and exercise medicine in undergraduate training. Lancet. 2012;380(9836):4–5. 10.1016/S0140-6736(12)60992-1. 22770445 10.1016/S0140-6736(12)60992-1

[27] Womersley K, Ripullone K. Medical schools should be prioritising nutrition and lifestyle education. Br J Sports Med. 2018;52(20):e6. 10.1136/bjsports-2018-j4861rep. ([published correction appears in Br J Sports Med. 2019 Mar;53(6):e3. 10.1136/bjsports-2018-j4861rep-corr1.]). 29650522 10.1136/bjsports-2018-j4861rep

[28] Matheson GO, Klügl M, Engebretsen L, et al. Prevention and management of noncommunicable disease: the IOC consensus statement, Lausanne 2013. Sports Med. 2013;43(11):1075–88. 10.1007/s40279-013-0104-3. 24129783 10.1007/s40279-013-0104-3

[29] Pandya T, Marino K. Embedding sports and exercise medicine into the medical curriculum; a call for inclusion. BMC Med Educ. 2018;18(1):306. 10.1186/s12909-018-1422-9. 30545345 10.1186/s12909-018-1422-9 PMC6293537

[30] Páez DC, Flórez J, Gómez MT, García D, Arango-Paternina CM, Duperly J. Curricular and pedagogical approaches for physical activity prescription training: a mixed-methods study of the “Exercise is Medicine” workshops in Colombia. BMC Med Educ. 2024;24(1):79. 10.1186/s12909-023-04999-3. 38254169 10.1186/s12909-023-04999-3 PMC10804704

[31] Matheson GO, Klügl M, Dvorak J, et al. Responsibility of sport and exercise medicine in preventing and managing chronic disease: applying our knowledge and skill is overdue. Br J Sports Med. 2011;45(16):1272–82. 10.1136/bjsports-2011-090328. 21948123 10.1136/bjsports-2011-090328

[32] World Health Organization. Framework for action on interprofessional education & collaborative practice. Geneva: WHO; 2010. WHO/HRH/HPN/10.3.

[33] Soan EJ, Street SJ, Brownie SM, Hills AP. Exercise physiologists: essential players in interdisciplinary teams for noncommunicable chronic disease management. J Multidiscip Healthc. 2014;7:65–8. 10.2147/JMDH.S55620. 24511238 10.2147/JMDH.S55620 PMC3913503

[34] Jones H, George KP, Scott A, et al. Charter to establish clinical exercise physiology as a recognised allied health profession in the UK: a call to action. BMJ Open Sport Exerc Med. 2021;7(3):e001158. 10.1136/bmjsem-2021-001158. 10.1136/bmjsem-2021-001158 PMC8458347 34631147

[35] Cook DA, Artino AR Jr. Motivation to learn: an overview of contemporary theories. Med Educ. 2016;50(10):997–1014. 10.1111/medu.13074. 27628718 10.1111/medu.13074 PMC5113774

[36] McCoy L, Pettit RK, Kellar C, Morgan C. Tracking active learning in the medical school curriculum: a learning-centered approach. J Med Educ Curric Dev. 2018;5:2382120518765135. 10.1177/2382120518765135. 29707649 10.1177/2382120518765135 PMC5912289

[37] Ericsson KA. Deliberate practice and acquisition of expert performance: a general overview. Acad Emerg Med. 2008;15(11):988–94. 10.1111/j.1553-2712.2008.00227.x. 18778378 10.1111/j.1553-2712.2008.00227.x

[38] Noormohammadpour P, Halabchi F, Mazaheri R, et al. Designing and implementing a curriculum for Sports and Exercise Medicine elective course for undergraduate medical students of Tehran University of Medical Sciences. Br J Sports Med. 2019;53(10):601–4. 10.1136/bjsports-2018-099462. 29934428 10.1136/bjsports-2018-099462

[39] Chatterjee R, Chapman T, Brannan MG, Varney J. GPs’ knowledge, use, and confidence in national physical activity and health guidelines and tools: a questionnaire-based survey of general practice in England. Br J Gen Pract. 2017;67(663):e668–75. 10.3399/bjgp17X692513. 28808077 10.3399/bjgp17X692513 PMC5604830

[40] Brauer DG, Ferguson KJ. The integrated curriculum in medical education: AMEE guide no. 96. Med Teach. 2015;37(4):312–22. 10.3109/0142159X.2014.970998. 25319403 10.3109/0142159X.2014.970998

[41] Chinese Society of Sports Science, Chinese Geriatrics Society, Wang Zhengzhen, et al. Guidelines for exercise prescriptions for sarcopenia. J Beijing Sport University, 2026, 49(01): 95-113. 10.19582/j.cnki.11-3785/g8.2026.01.008.

[42] [1] Xu Kun, Cheng Jingwei, Wang Congshuai, et al. The role of exercise in the management of metabolic associated fatty liver disease: interpretation of international expert consensus by the american college of sports medicine and the Australian Society for Exercise and Sports Sciences [J/OL]. Chinese General Practice, 1–9 [2026–03–02]. https://link.cnki.net/urlid/13.1222.R.20251226.1646.004.

[43] Song Y, Wang J, Chen X, Guo Y, Wang X, Liang W. Facilitators and barriers to exercise influenced by traditional Chinese culture: a qualitative study of Chinese patients undergoing hemodialysis. J Transcult Nurs. 2019;30(6):558–68. 10.1177/1043659618823908. 30702029 10.1177/1043659618823908

[44] Composing and editorial board of physical activity guidelines for Chinese. Zhonghua Yu Fang Yi XueZaZhi. 2022;56(1):7-8. 10.3760/cma.j.cn112150-20211119-01070 10.3760/cma.j.cn112150-20211119-01070 35092983

[45] World Health Organization. Seventy-fifth World Health Assembly: Resolutions and decisions, annexes (WHA75/2022/REC/1). Geneva: World Health Organization; 2022. https://apps.who.int/gb/ebwha/pdf_files/WHA75-REC1/A75_REC1_Interactive_en.pdf.

[46] United Nations General Assembly. Transforming our world: the 2030 Agenda for Sustainable Development (A/RES/70/1). New York: United Nations; 2015.

[47] Young A, Gray JAM, Ennis JR. ‘Exercise medicine’: the knowledge and beliefs of final-year medical students in the United Kingdom. Med Educ. 1983;17(6):327–32. 10.1111/j.1365-2923.1983.tb01122.x. 10.1111/j.1365-2923.1983.tb01122.x 6633308

