# Supplementary material for: Integration of exercise and sports medicine curriculum in China: a structured pilot course evaluation conducted among medical students
Source: BMC Med Educ. 2026 May 9;26:1049. doi: 10.1186/s12909-026-09013-0 (PMC13326290; doi:10.1186/s12909-026-09013-0)
Supplement: Supplementary file 1 — Supplementary Material 1. [file 12909_2026_9013_MOESM1_ESM.pdf]

Survey on the Learning Outcomes of the Sports and Exercise Medicine

Preliminary Questions

1. Are you willing to participate in this survey?
2. Which school/college are you currently enrolled in?
3. What is your email address?
4. What is your current year of study?
5. What is your gender?
6. Do you have prior experience in sports science or sports and exercise medicine (SEM)? For example, holding a relevant degree (e.g., Bachelor's in Sport Science), personal trainer certification, or participation in exercise prescription/clinical exercise physiology training.

Section I. Knowledge Dimension

For the following statements, please indicate your level of agreement on a 5-point Likert scale:

1 = Strongly Disagree, 2 = Disagree, 3 = Neutral, 4 = Agree, 5 = Strongly Agree.

K1. I understand that physical activity is important for disease prevention and management.

K2. I understand that physical activity is important for disease treatment.

K3. I am familiar with the key content of the national physical activity guidelines issued in China.

K4. Providing patients with advice on physical activity is an essential responsibility of physicians.

K5. I am familiar with the essential components of an exercise prescription (frequency, intensity, type, time, progression, and periodization) and their application.

K6. I understand the precautions and contraindications of exercise for patients with common diseases and can identify risk factors to ensure safe guidance.

Section II. Skills Dimension

S1. I am able to systematically obtain a patient's physical activity history and assess their daily activity levels.

S2. I am capable of developing individualized exercise prescriptions for patients under different conditions (including frequency, intensity, type, and time).

S3. I can assess contraindications and safety concerns when prescribing exercise for patients with chronic diseases or special conditions.

S4. I am able to demonstrate basic exercise techniques and instruct patients in correct movement patterns.

S5. I can apply behavioral change techniques (e.g., motivational interviewing) to encourage patients to adhere to exercise programs.

S6. I know how to evaluate whether a patient's physical activity level has achieved measurable health benefits.

Section III. Confidence Dimension

C1. I am confident in providing effective counseling on physical activity for various patients and addressing their concerns.

C2. I am confident in offering exercise-related advice to patients.

C3. I am confident in designing safe and effective exercise prescriptions even for patients with complex chronic diseases.

C4. I am confident in integrating physical activity advice into clinical practice even under time constraints or when patient adherence is low.

C5. I am confident in answering patients' questions about exercise and correcting their misconceptions.

C6. I am confident that in my future medical career, I will consistently incorporate exercise promotion into routine practice.

Section IV. Motivation and Attitudes Dimension

M1. I believe that helping patients improve their physical activity levels is an important responsibility of healthcare professionals.

M2. I believe that the role of regular physical activity in disease prevention and rehabilitation is as important as pharmacological treatment.

M3. I intend to apply the knowledge and skills learned in this course to future medical practice (e.g., routinely providing exercise advice or prescriptions to patients).

M4. This course has stimulated my interest in sports and exercise medicine, and I plan to further study exercise-related health promotion after the course.

M5. As a future healthcare professional, I am willing to set an example by maintaining an active

lifestyle to influence and motivate my patients.

M6. As a future healthcare professional, I am willing to share exercise-related knowledge with colleagues and encourage them to strengthen their expertise in this area.

Section V. Course Evaluation

E1. Through this SEM course, my knowledge and practical skills in this field have improved.

E2. The objectives, content, and knowledge delivered in this course were clear.

E3. The overall course structure and design were highly reasonable.

E4. The content of the course was relevant and useful to the intended learning objectives.

E5. I would like to see similar courses offered in the future.
