# Supplementary material for: Integration of exercise and sports medicine curriculum in China: a structured pilot course evaluation conducted among medical students
Source: BMC Med Educ. 2026 May 9;26:1049. doi: 10.1186/s12909-026-09013-0 (PMC13326290; doi:10.1186/s12909-026-09013-0)
Supplement: Supplementary file 2 — Supplementary Material 2. [file 12909_2026_9013_MOESM2_ESM.pdf]

SEM Curriculum Examination Papers (Versions A and B)

Multiple Choice Questions: 40 questions, 2 points each (total 80 points)

Practical Questions: 20 points

Total: 100 points

Exam Paper A

1. Which of the following options is consistent with the Chinese physical activity guidelines for children under 2 years of age?

A. Children who can walk independently should engage in at least 180 minutes of physical activity per day.

B. Children who can walk independently should engage in at least 180 minutes of physical activity per week.

C. Children who can walk independently should engage in physical activity at least 5 times per week, each session no less than 180 minutes.

D. Children who can walk independently should engage in physical activity at least 3 times per week, each session no less than 180 minutes.

Answer: A

2. Which of the following options is consistent with the Chinese physical activity guidelines for children aged 3–5 years?

A. At least 180 minutes of physical activity daily, sedentary time no more than 1 hour.

B. At least 180 minutes of physical activity weekly, including 60 minutes of energetic play, sedentary time no more than 1 hour.

C. At least 5 times per week, each session at least 180 minutes including 60 minutes of energetic play, sedentary time no more than 1 hour.

D. At least 3 times per week, each session at least 180 minutes including 60 minutes of energetic play, sedentary time no more than 1 hour.

Answer: A

3. Which of the following options is consistent with the Chinese physical activity guidelines for children and adolescents aged 6–17 years?

A. At least 60 minutes of physical activity daily, sedentary time no more than 1 hour.

B. At least 60 minutes of moderate or higher intensity physical activity daily, sedentary time no more than 1 hour.

C. At least 60 minutes of moderate or higher intensity physical activity daily, plus at least 3 sessions of muscle-strengthening exercises weekly, sedentary time no more than 1 hour.

D. 3–5 times per week, each session at least 60 minutes, plus at least 3 sessions of muscle-strengthening exercises weekly, sedentary time no more than 1 hour.

Answer: C

4. Which of the following options is consistent with the Chinese physical activity guidelines for adults aged 18–64 years?

A. 150–300 minutes of moderate-intensity or 75–150 minutes of vigorous-intensity aerobic activity per week, sedentary time no more than 1 hour.

B. 150–300 minutes of moderate-intensity or 75–150 minutes of vigorous-intensity aerobic activity per week, plus at least 2 sessions of strength training per week.

C. 150–300 minutes of moderate-intensity or 75–150 minutes of vigorous-intensity aerobic activity per week, plus at least 3 sessions of muscle-strengthening exercises per week, sedentary time no more than 1 hour.

D. 3–5 times per week, each session at least 60 minutes of aerobic activity, plus at least 3 sessions of muscle-strengthening exercises per week, sedentary time no more than 1 hour.

Answer: B

5. Which of the following options is consistent with the Chinese physical activity guidelines for adults aged 65 years and above?

A. 150–300 minutes of moderate-intensity or 75–150 minutes of vigorous-intensity aerobic activity per week, sedentary time no more than 1 hour.

B. 150–300 minutes of moderate-intensity or 75–150 minutes of vigorous-intensity aerobic activity per week, plus at least 2 sessions of strength training per week.

C. 150–300 minutes of moderate-intensity or 75–150 minutes of vigorous-intensity aerobic activity per week, along with balance, flexibility, and mobility exercises, increasing physical activity as much as possible.

D. 3–5 times per week, each session at least 60 minutes of aerobic activity, along with balance, flexibility, and mobility exercises, increasing physical activity as much as possible.

Answer: C

6. What is the minimum recommended weekly duration of moderate-intensity physical activity in an exercise prescription?

A. 75 minutes

B. 150 minutes

C. 200 minutes

D. 250 minutes

Answer: B

7. Which of the following is NOT a key component of an exercise prescription?

A. Exercise frequency

B. Exercise intensity

C. Exercise type

D. Exercise preference

Answer: D

8. In the FITT principle, "I" stands for:

A. Frequency

B. Intensity

C. Time

D. Type

Answer: B

9. What is the recommended frequency of muscle-strengthening activities?

A. Daily

B. At least once per week

C. At least twice per week

D. At least three times per week

Answer: C

10. Physical inactivity is NOT associated with increased risk of which of the following diseases?

A. Type 2 diabetes

B. Coronary heart disease

C. Osteoporosis

D. Myopia

Answer: D

11. What is the commonly recommended minimum daily step goal?

A. 5,000 steps

B. 7,000 steps

C. 10,000 steps

D. 15,000 steps

Answer: C

12. Which of the following indicators can be used to measure exercise intensity?

A. Heart rate

B. Rating of perceived exertion

C. Metabolic equivalent (MET)

D. All of the above

Answer: D

13. In pre-exercise health screening tools, the primary function of PAR-Q is to:

A. Assess fitness level

B. Assess disease risk

C. Assess dietary habits

D. Assess psychological status

Answer: B

14. Target heart rate in exercise prescriptions is generally recommended to be within what percentage of maximum heart rate?

A. 20%–40%

B. 40%–60%

C. 60%–80%

D. 80%–100%

Answer: C

15. According to physical activity guidelines, what is the minimum recommended frequency of aerobic exercise per week?

A. Once

B. Twice

C. Three times

D. Five times

Answer: C

16. Which of the following is NOT necessary when collecting an exercise history?

A. Patient’s exercise preference

B. Patient’s family economic status

C. Patient’s past exercise experience

D. Patient’s barriers to exercise

Answer: B

17. Which strategy is most appropriate for enhancing patient exercise motivation?

A. Emphasizing exercise risks

B. Setting unrealistic goals

C. Solution-focused communication

D. Avoiding goal-setting

Answer: C

18. Which of the following patients should start an exercise program with caution?

A. Healthy young adults

B. Patients who recently underwent cardiac surgery

C. Elderly individuals with exercise habits

D. Adults with normal BMI

Answer: B

19. Which of the following is NOT required in an exercise prescription?

A. Exercise frequency

B. Exercise intensity

C. Specific exercise type

D. Patient’s income

Answer: D

20. How many times per week should a typical adult engage in strength training?

A. Once

B. Twice

C. Four times

D. Daily

Answer: B

21. Which type of activity is recommended for obese patients beginning an exercise program?

A. High-intensity anaerobic exercise

B. Low-impact aerobic exercise

C. Sprinting

D. High-intensity interval training (HIIT)

Answer: B

22. Appropriate exercise intensity for patients with hypertension corresponds to what percentage of maximum heart rate?

A. 40%–60%

B. 50%–70%

C. 70%–80%

D. 80%–90%

Answer: A

23. Patients with heart failure should avoid which of the following?

A. Moderate aerobic exercise

B. Strength training

C. High-intensity anaerobic exercise

D. Gentle stretching

Answer: C

24. Which exercise mode is preferred for patients with peripheral arterial disease?

A. Vigorous running

B. Long-distance cycling

C. Intermittent walking

D. Heavy resistance training

Answer: C

25. For patients with type 2 diabetes, which exercise type is recommended?

A. High-intensity interval training

B. Combined resistance and aerobic training

C. Sprinting

D. Extreme endurance training

Answer: B

26. Recommended exercise intensity for patients with chronic liver disease is:

A. Moderate-intensity aerobic exercise

B. Long-distance running

C. Maximal weightlifting

D. High-intensity interval training

Answer: A

27. For patients with chronic kidney disease, which of the following is correct?

A. Exercise as vigorously as possible

B. Strictly control exercise intensity, monitor disease progression

C. Engage in prolonged endurance training

D. Perform short-duration, intense exercise

Answer: B

28. Key focus in exercise prescription for breast cancer patients is:

A. Enhancing explosive power

B. Preventing lymphedema and gradually increasing intensity

C. Training at the fastest possible speed

D. Extreme resistance training

Answer: B

29. Patients with respiratory diseases are best suited for:

A. Marathon running

B. Low-to-moderate intensity interval training

C. Weightlifting and high-intensity training

D. Extreme sports

Answer: B

30. When designing exercise prescriptions for patients with mental disorders, the priority should be:

A. High-intensity long-distance running

B. Fun and sustainability of exercise

C. Primarily weightlifting

D. Anaerobic explosive training

Answer: B

Part II. Multiple-Response Questions (10 questions, 2 points each, total 20 points; includes case questions)

31. For an osteoporosis patient starting an exercise program, which factors should be considered?

A. Avoid high-impact activities

B. Recommend resistance training

C. Avoid all exercise

D. Emphasize balance training

Answer: A, B, D

32. Which are the key elements generally included in an exercise prescription?

A. Frequency

B. Intensity

C. Time

D. Type

Answer: A, B, C, D

33. In which situations should pre-exercise health risk assessment be performed?

A. Recent surgery

B. Family history of disease

C. Smoking

D. Age above 65 years

Answer: A, B, C, D

34. In designing exercise prescriptions for breast cancer patients, which considerations are important?

A. Avoid overexertion

B. Monitor risk of lymphedema

C. Gradually increase intensity

D. Recommend vigorous exercise

Answer: A, B, C

35. Key considerations in designing exercise prescriptions for older adults include:

A. Emphasizing balance training

B. Recommending high-intensity exercise

C. Avoiding resistance training

D. Paying attention to cardiovascular safety

Answer: A, D

Case Questions (Questions 36–40)

36. For a patient with type 2 diabetes, which considerations are important in exercise planning?

A. Regular blood glucose monitoring

B. Moderate resistance training

C. High intake of sugary drinks

D. Moderate-intensity aerobic exercise

Answer: A, B, D

37. For a patient with depression, which considerations are important in exercise prescription?

A. Exercise safety

B. Social interaction

C. Enjoyable activities

D. High-risk sports

Answer: A, B, C

38. For a patient with hypertension, which considerations are important in exercise design?

A. Monitor blood pressure before exercise

B. Avoid vigorous exercise

C. Regularly assess cardiovascular risk

D. Recommend high-intensity vigorous exercise

Answer: A, B, C

39. For a 60-year-old patient with heart failure, which activities should be avoided?

A. Explosive exercises

B. Prolonged exercise sessions

C. Moderate, short-duration exercise

D. Excessive resistance training

Answer: A, B, D

40. Characteristics of exercise prescriptions for patients with peripheral arterial disease include:

A. Intermittent walking training

B. Monitoring limb symptoms

C. Avoiding overexertion

D. Recommending long-distance, high-speed cycling

Answer: A, B, C

Exam Paper B

1. Which of the following options is consistent with the Chinese physical activity guidelines for children under 2 years of age?

A. Children who can walk independently should engage in at least 180 minutes of physical activity per day.

B. Children who can walk independently should engage in at least 180 minutes of physical activity per week.

C. Children who can walk independently should engage in physical activity at least 5 times per week, each session no less than 180 minutes.

D. Children who can walk independently should engage in physical activity at least 3 times per week, each session no less than 180 minutes.

Answer: A

2. Which of the following options is consistent with the Chinese physical activity guidelines for children aged 3–5 years?

A. At least 180 minutes of physical activity daily, sedentary time no more than 1 hour.

B. At least 180 minutes of physical activity weekly, including 60 minutes of energetic play, sedentary time no more than 1 hour.

C. At least 5 times per week, each session at least 180 minutes including 60 minutes of energetic play, sedentary time no more than 1 hour.

D. At least 3 times per week, each session at least 180 minutes including 60 minutes of energetic play, sedentary time no more than 1 hour.

Answer: A

3. Which of the following options is consistent with the Chinese physical activity guidelines for children and adolescents aged 6–17 years?

A. At least 60 minutes of physical activity daily, sedentary time no more than 1 hour.

B. At least 60 minutes of moderate or higher intensity physical activity daily, sedentary time no more than 1 hour.

C. At least 60 minutes of moderate or higher intensity physical activity daily, plus at least 3 sessions of muscle-strengthening exercises weekly, sedentary time no more than 1 hour.

D. 3–5 times per week, each session at least 60 minutes, plus at least 3 sessions of muscle-strengthening exercises weekly, sedentary time no more than 1 hour.

Answer: C

4. Which of the following options is consistent with the Chinese physical activity guidelines for adults aged 18–64 years?

A. 150–300 minutes of moderate-intensity or 75–150 minutes of vigorous-intensity aerobic activity per week, sedentary time no more than 1 hour.

B. 150–300 minutes of moderate-intensity or 75–150 minutes of vigorous-intensity aerobic activity per week, plus at least 2 sessions of strength training per week.

C. 150–300 minutes of moderate-intensity or 75–150 minutes of vigorous-intensity aerobic activity per week, plus at least 3 sessions of muscle-strengthening exercises per week, sedentary time no more than 1 hour.

D. 3–5 times per week, each session at least 60 minutes of aerobic activity, plus at least 3 sessions of muscle-strengthening exercises per week, sedentary time no more than 1 hour.

Answer: B

5. Which of the following options is consistent with the Chinese physical activity guidelines for adults aged 65 years and above?

A. 150–300 minutes of moderate-intensity or 75–150 minutes of vigorous-intensity aerobic activity per week, sedentary time no more than 1 hour.

B. 150–300 minutes of moderate-intensity or 75–150 minutes of vigorous-intensity aerobic activity per week, plus at least 2 sessions of strength training per week.

C. 150–300 minutes of moderate-intensity or 75–150 minutes of vigorous-intensity aerobic activity per week, along with balance, flexibility, and mobility exercises, increasing physical activity as much as possible.

D. 3–5 times per week, each session at least 60 minutes of aerobic activity, along with balance,

flexibility, and mobility exercises, increasing physical activity as much as possible.

Answer: C

6. What is the minimum recommended weekly duration of moderate-intensity physical activity in an exercise prescription?

A. 75 minutes

B. 150 minutes

C. 200 minutes

D. 250 minutes

Answer: B

7. Which of the following is NOT a key component of an exercise prescription?

A. Exercise frequency

B. Exercise intensity

C. Exercise type

D. Dietary nutrition

Answer: D

8. In the FITT principle, "I" stands for:

A. Frequency

B. Intensity

C. Time

D. Type

Answer: B

9. What is the recommended frequency of muscle-strengthening activities?

A. Daily

B. At least once per week

C. At least twice per week

D. At least three times per week

Answer: C

10. Physical inactivity is NOT associated with increased risk of which of the following diseases?

A. Type 2 diabetes

B. Coronary heart disease

C. Osteoporosis

D. Myopia

Answer: D

11. What is the commonly recommended minimum daily step goal?

A. 5,000 steps

B. 7,000 steps

C. 10,000 steps

D. 15,000 steps

Answer: C

12. Which of the following indicators can be used to measure exercise intensity?

A. Heart rate

B. Rating of perceived exertion

C. Metabolic equivalent (MET)

D. All of the above

Answer: D

13. In pre-exercise health screening tools, the primary function of PAR-Q is to:

A. Assess fitness level

B. Assess disease risk

C. Assess dietary habits

D. Assess psychological status

Answer: B

14. Target heart rate in exercise prescriptions is generally recommended to be within what percentage of maximum heart rate?

A. 20%–40%

B. 40%–60%

C. 60%–80%

D. 80%–100%

Answer: C

15. According to physical activity guidelines, what is the minimum recommended frequency of aerobic exercise per week?

A. Once

B. Twice

C. Three times

D. Five times

Answer: C

16. Which of the following is NOT necessary when collecting an exercise history?

A. Patient's exercise preference

B. Patient's family economic status

C. Patient's past exercise experience

D. Patient's barriers to exercise

Answer: B

17. Which strategy is most appropriate for enhancing patient exercise motivation?

A. Emphasizing exercise risks

B. Setting unrealistic goals

C. Solution-focused communication

D. Avoiding goal-setting

Answer: C

18. Which of the following patients should start an exercise program with caution?

A. Healthy young adults

B. Patients who recently underwent cardiac surgery

C. Elderly individuals with exercise habits

D. Adults with normal BMI

Answer: B

19. Which of the following is NOT required in an exercise prescription?

A. Exercise frequency

B. Exercise intensity

C. Specific exercise type

D. Patient’s income

Answer: D

20. How many times per week should a typical adult engage in strength training?

A. Once

B. Twice

C. Four times

D. Daily

Answer: B

21. For which patient group is the lowest exercise intensity recommended?

A. Hypertension

B. Osteoporosis

C. Heart failure

D. Obesity

Answer: C

22. For patients with severe osteoarthritis, which form of exercise is least recommended?

A. Aquatic walking

B. Cycling

C. Jogging

D. Stretching

Answer: C

23. When breast cancer patients engage in resistance training, which should be most avoided?

A. Light upper limb resistance

B. Rapid increase of training intensity

C. Lower limb resistance training

D. Post-training relaxation

Answer: B

24. Which training mode is appropriate for patients with hypertension?

A. Heavy weightlifting

B. Low-weight, high-repetition strength training

C. Explosive heavy resistance training

D. Sprint training

Answer: B

25. Which indicators should be monitored in exercise training for patients with chronic kidney disease?

A. Blood glucose

B. Oxygen saturation

C. Creatine kinase

D. Blood pressure and renal function markers

Answer: D

26. For an obese patient beginning an exercise program, which activity is most appropriate?

A. Long-distance running

B. High-intensity interval training

C. Low-impact aerobic exercise

D. Vigorous jumping exercises

Answer: C

27. What is the target RPE (Rating of Perceived Exertion) for heart failure patients during exercise?

A. 5–7

B. 11–14

C. 16–18

D. 19–20

Answer: B

28. Which of the following is NOT an absolute contraindication for pre-exercise screening?

A. Unstable angina

B. Acute myocardial infarction

C. Uncontrolled arrhythmia

D. Stable hypertension

Answer: D

29. Which exercise mode is most suitable for patients with peripheral arterial disease?

A. Intermittent walking

B. Fast cycling

C. Vigorous jumping

D. Marathon running

Answer: A

30. Which parameter should be closely monitored in patients with respiratory diseases during exercise?

A. Oxygen saturation

B. Blood glucose

C. Lactate threshold

D. Muscle endurance

Answer: A

Part II. Multiple-Response Questions (10 questions, 2 points each, total 20 points)

31. Case: A 55-year-old male patient with severe osteoarthritis. Which exercise methods are recommended?

A. Aquatic walking or hydrotherapy

B. Low-intensity cycling

C. High-impact activities (e.g., basketball, volleyball)

D. Gentle stretching and flexibility exercises

Answer: A, B, D

32. Which are the key elements generally included in an exercise prescription?

A. Frequency

B. Intensity

C. Time

D. Type

Answer: A, B, C, D

33. In which situations should pre-exercise health risk assessment be performed?

A. Recent surgery

B. Family history of disease

C. Smoking

D. Age above 65 years

Answer: A, B, C, D

34. In designing exercise prescriptions for breast cancer patients, which considerations are important?

A. Avoid overexertion

B. Monitor risk of lymphedema

C. Gradually increase intensity

D. Recommend vigorous exercise

Answer: A, B, C

35. Key considerations in designing exercise prescriptions for older adults include:

A. Emphasizing balance training

B. Recommending high-intensity exercise

C. Resistance training

D. Paying attention to cardiovascular safety

Answer: A, D

36. Case: A patient with chronic liver disease. Which considerations should be made in the exercise plan?

A. Select moderate-intensity aerobic exercise

B. Avoid vigorous exercise

C. Regularly monitor liver function

D. Encourage high-intensity strength training

Answer: A, B, C

37. For a patient with peripheral arterial disease, which considerations are correct during training?

A. Take regular breaks to relieve pain

B. Gradually increase training intensity

C. Ignore leg pain and continue exercising

D. Monitor post-exercise symptom recovery

Answer: A, B, D

38. Which exercise recommendations are correct for patients with chronic kidney disease?

A. Regular renal function monitoring

B. High-intensity training to improve renal function

C. Moderate-intensity aerobic exercise

D. Monitor blood pressure changes

Answer: A, C, D

39. Key safety considerations for older adults in exercise prescriptions include:

A. Balance training

B. Explosive power training

C. Flexibility training

D. Minimizing fall risk during exercise

Answer: A, C, D

40. For a patient with depression, which strategies help improve exercise adherence?

A. Set easily achievable initial goals

B. Choose enjoyable exercise activities

C. Encourage training alone to avoid social interaction

D. Gradually increase exercise intensity

Answer: A, B, D

Practical Examination: Exercise History Taking and Exercise Prescription Design (20 points)

I. Objective

To assess students' comprehensive ability to obtain a patient's exercise history, conduct health risk assessment, and develop an individualized exercise prescription.

II. Procedure

1. Exercise History Taking (5 minutes)

Students communicate with a standardized patient (SP; presenting with any chronic disease).

Complete collection of exercise-related history, including:

Previous exercise history

Current exercise status

Health status (medical history, family history)

Physical activity preferences and barriers

Lifestyle factors (e.g., diet, smoking, etc.)

2. Health Risk Assessment (5 minutes)

Based on the collected history, complete a pre-exercise health risk screening form (e.g., PAR-Q).

Determine whether the patient has exercise-related risks and whether further medical evaluation is required.

3. Exercise Prescription Design (5 minutes)

Develop an individualized exercise prescription according to the patient's history and risk assessment results.

Explicitly define the FITT principle:

Frequency

Intensity

Time

Type

Clearly explain the rationale for the prescription and specify safety precautions.

III. Scoring Criteria

Completeness of history taking and communication skills: 6 points

Accuracy of risk assessment: 6 points

Appropriateness and individualization of exercise prescription design: 8 points

Total: 20 points
